# Unusual presentation of primary T-cell lymphoblastic lymphoma: description of two cases

**DOI:** 10.1186/1746-1596-9-124

**Published:** 2014-06-20

**Authors:** Maria R Ambrosio, Monica Onorati, Bruno J Rocca, Alessandro Ginori, Giuseppe Lobello, Guido Petracco, Alessandro Davide Videtta, Franca Di Nuovo, Rosa Santopietro, Stefano Lazzi

**Affiliations:** 1 Department of Medical Biotechnologies, Section of Pathology, University of Siena, Siena, Italy; 2 Section of Pathology, G. Salvini Hospital, Garbagnate Milanese, Italy; 3 Section of Pathology, Ospedale di Circolo di Busto Arsizio, Presidio Ospedaliero di Saronno, Saronno, Italy; 4 Section of Pathology, Azienda Ospedaliera Universitaria Senese, Siena, Italy

**Keywords:** Lymphoblastic lymphoma, TdT, molecular biology

## Abstract

**Abstract:**

**Background**

T-cell lymphoblastic lymphoma comprises approximately 85-90% of all lymphoblastic lymphomas. It often arises as a mediastinal mass, and with bone marrow involvement. Presentation at other sites without nodal or mediastinal localization is uncommon.

**Case report**

We describe clinical, histologic, immunohistochemical, and molecular features of two cases of primary T-cell lymphoblastic lymphoma arising respectively in uterine corpus and testis. The tumors were composed by medium to large cells, exhibiting a diffuse pattern of growth but sometimes forming indian files or pseudo-rosettes. The neoplastic cells strongly expressed TdT and T-cell markers in both uterine corpus and testis. However, the testis case also showed aberrant expression of B-cell markers, thus molecular biology was necessary to achieve a final diagnosis. T-cell receptor gene rearrangement analysis identified a T-cell origin.

**Conclusions**

To the best of our knowledge, only one doubtful previous case of primary uterine T-cell lymphoblastic lymphoma and no previous cases of primary testicular T-cell lymphoblastic lymphoma have been reported. Due to the morphology of neoplastic cells, a challenging differential diagnosis with all the tumors belonging to the so-called small round blue cell tumor category is mandatory. In ambiguous lineage cases, molecular biology may represent an adequate tool to confirm diagnosis.

**Virtual Slides:**

The virtual slide(s) for this article can be found here: http://www.diagnosticpathology.diagnomx.eu/vs/1559880973128230

## Background

Precursor lymphoid neoplasms include acute lymphoblastic leukemias (ALLs) and lymphoblastic lymphomas (LBLs) of either B- or T-cell origin [1].

According to the last World Health Organization (WHO) classification of Tumors of Haematopoietic and Lymphoid Tissues, T-cell ALL/LBL is a neoplasm of lymphoblasts committed to the T-cell lineage involving bone marrow and blood (T-ALL) [2].

T-LBL comprises approximately 85-90% of all lymphoblastic lymphomas; similarly to its leukemic counterpart, it is most frequent in males and in late childhood, constituting only a small percentage of adult cases [3]. T-LBL usually presents as a mediastinal mass, and with bone marrow localization. Skin, tonsil, liver, spleen, central nervous system (CNS) and testis in males may be affected, although presentation at these sites without nodal or mediastinal involvement is uncommon [2]. The lymphoblasts in T-ALL/LBL (small to medium-sized cells with scant cytoplasm, convoluted or round nuclear contours, high nuclear/cytoplasmic ratio, immature nuclear chromatin with usual inconspicuous nucleoli) are morphologically indistinguishable from those of B-ALL/LBL. The neoplastic cells express terminal deoxynucleotidyl transferase (TdT), CD34, CD99 and variable CD2, CD3, CD4, CD5, CD7, CD8 [2].

Treatment is generally divided into three phases employed by using different drugs: induction (dexamethasone, prednisone or prednisolone, vincristine, asparaginase and/or doxorubicin), consolidation (high dose methotrexate plus mercaptopurine, high-dose asparaginase or reinduction), maintenance (weekly methotrexate plus daily mercaptopurine) [4-6]. Given that standard doses of chemotherapy may not reach leukemia cells in brain and spinal cord, the cells are able to find sanctuary in the CNS, especially for cases with testicular involvement. Therefore, another important therapeutic strategy to prevent CNS relapse is prophylaxis by intrathecal injection [1]. Unlike to ALL and B-cell LBL, there are no clear prognostic factors that may predict remission or survival in T-cell LBL, although it frequently occurs in older patients showing high white blood cell count, both features associated with an adverse clinical course [1]. It has been recently demonstrated that a treatment strategy that includes planner consolidation with stem-cell transplantation (SCT) produces long-term outcome in selected adult patients [4,6]. The main differential diagnoses of LBL include Burkitt lymphoma (BL), diffuse large-B cells lymphoma (DLBCL), blastic variant of mantle cell lymphoma (MCL), small lymphocytic lymphoma, B1 thymoma, acute myeloid leukaemia, myeloid sarcoma, small round blue cell tumors (including Ewing sarcoma-ES/peripheral neuroectodermal tumour-PNET, neuroblastoma, embryonal rhabdomyosarcoma, medulloblastoma) [1,6,7].

We describe two cases of primary T-LBL arising in atypical sites, respectively uterine corpus and testis. The importance of differential diagnosis with other lymphoid and non lymphoid neoplasms is underlined.

## Cases presentation

### Case 1

**Figure 1:**
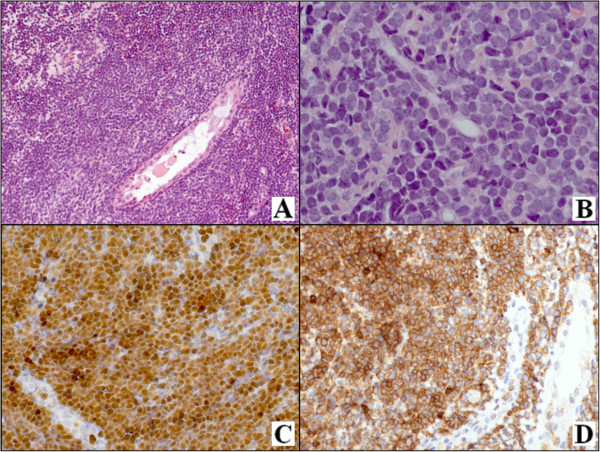
**Histological and immunohistochemical features of uterine T-cell lymphoblastic lymphoma.** A diffuse proliferation of round blue cells infiltrating endometrial glands and stroma and dissecting the muscle fiber is observed **(A)**. Cells exhibit a blastic appearance **(B)**. They are positive for TdT **(C)** and CD2 **(D)**. [A-B: haematoxylin and eosin (H & E), C: TdT stain, D: CD2 stain; A, C-D: original magnification (O.M.), 20×, B: O.M., 40×].

A 64 years-old female was admitted to Siena University Hospital for persistent vaginal bleeding. Physical examination revealed marked enlargement of the uterus, and abdominal ultrasonography showed a 6.0x4.0 cm hypoechoic mass in uterine corpus. Endometrial biopsy was performed. The surgical specimen consisted of three brownish fragments ranging from 0,2 to 0,7 cm in maximum diameter. Histological examination of the formalin fixed paraffin-embedded sections showed a polypoid lesion with atrophic endometrium. On this background, a diffuse proliferation of round blue cells infiltrating endometrial glands and stroma, and dissecting the muscle fibres was observed (Figure 1A). Cells were medium to large and exhibit a diffuse pattern of growth sometimes forming indian files or pseudo-rosettes (Figure 1B). A vaguely starry-sky appearance was also present. Cytologically, some cells showed scant cytoplasm, dense nuclear chromatin and multiple inconspicuous nucleoli, whereas other demonstrated prominent nucleoli. There were numerous mitotic figures. Neoplastic cells were positive for TdT (Figure 1C), CD2 (Figure 1D), CD7, CD3, CD10. Proliferative index (Ki-67) was high (about 90%). Molecular studies evidenced a clonal T-cell receptor (TCR) gene rearrangement. Diagnosis of T-LBL was made.

The patient underwent bone marrow biopsy with negative results and complete staging evaluation with whole body computed tomography (CT)-scan revealed that the disease was limited to the uterine corpus.

Treatment with systemic cyclophosphamide, vincristine sulfate, adryamicin, dexamethasone (hyper-CVAD) protocol together with intrathecal chemotherapy started. The patient died after eighteen months follow-up for a pulmonary infection.

### Case 2

**Figure 2:**
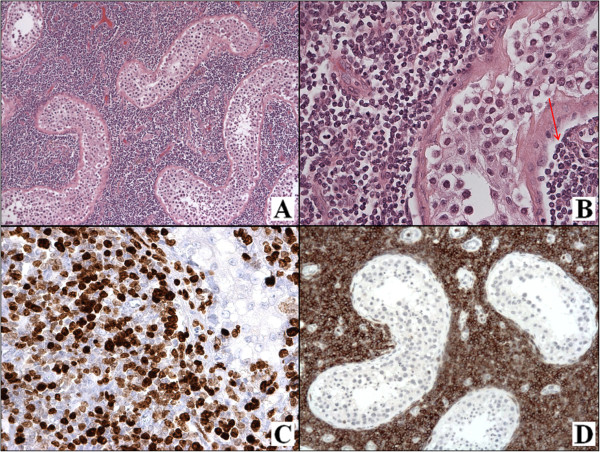
**Histological and immunohistochemical findings in T-cell lymphoblastic lymphoma of the testis.** The tubules are separated by a proliferation of blue uniform cells with an interstitial pattern of growth, occasionally invading the lining epithelium **(A-B)**. The neoplastic population expresses TdT **(C)** and CD2 **(D)** (A-B: H & E, C: TdT stain, D: CD2 stain; A: O.M., 5×; B, D: O.M., 20×; C: O.M., 40×).

A 38-year-old male presented to Garbagnate Milanese Hospital for a sudden unilateral enlargement of the scrotum. His past medical history was unremarkable. On physical examination, a painful solid mass of the left testis was observed. The ultrasound sonography showed a highly vascularized, hypoechoic lesion, completely infiltrating the testis. The clinical diagnosis was testicular neoplasm and a total orchiectomy was performed.On gross examination, a complete replacement of the testis by a fleshy, whitish, homogenous mass infiltrating the para-testicular tissues was observed. Microscopically, an extensive infiltrate of blue uniform cells between and displacing seminiferous tubules was noted. The neoplastic cells presented an interstitial pattern of growth, surrounding and focally infiltrating the lining epithelium of seminiferous tubules (Figure 2A-B). High endothelial venules were also observed. The neoplastic population typically grew in indian file (Figure 2B arrow) and showed a lymphoid appearance with morphologic features of a precursor lymphoma. They consisted of small to medium sized cells with round to oval nuclei, sometimes convoluted, with dispersed nuclear chromatin, inconspicuous nucleoli, and scanty, faintly basophilic cytoplasm. Mitoses were frequent. The neoplastic population expressed TdT (Figure 2C), and both T- (Figure 2D) and B-cell markers. The proliferative index (Ki-67) was high (about 95%). As the lineage was ambiguous due to the positivity to CD2, CD7, Pax-5 and CD79a, molecular biology was carried out, showing a clonal rearrangement of the gamma TCR gene.

Bone marrow biopsy was negative, whole body CT-scan showed neither lymphoadenopathies nor mediastinal mass, thus a diagnosis of primary T-LBL was made. The patient was treated with hyper-CVAD protocol associated to systemic and intrathecal injection of drugs plus high-dose cytarabine and methotrexate. At the last follow-up (27 months after the diagnosis) the patient is alive and under consolidation therapy.

## Discussion

In the last WHO classification, T-cell LBL is considered an immature malignancy, thought to be the nodal/extra-nodal presentation of ALL [1,2]. Most patients are adolescent or young adults who present with mediastinal mass and bone marrow localization [8-10]. Although rarely, the tumor may involve lymph nodes and extra-nodal sites (spleen, liver, testis and CNS) [2]. However, its occurrence as a primary tumor of the reproductive system is uncommon and rarely described in literature [11-14].

The peculiarities of our cases are multiple: the site of presentation (uterus and testis), the absence of bone marrow involvement, the age of the patients (64 and 38 years respectively), the ambiguous lineage of the neoplastic cells at immunohistochemistry in one case. In addition, none of our patients developed a leukemic disease at the last follow-up (18 months in the first case and 27 months in the second case).

The criteria to assess the primitivity of a LBL establish that diagnosis is correct only if the disease is confined to the organ and no signs of leukemia are present at diagnosis or develop during follow-up [15,16]. Therefore, our cases may be considered as primary T-LBL. To the best of our knowledge, only one previous case of T-LBL of the uterus was described in the literature, occurring in a 25 years old female [11]. However, the lack of a complete immunohistochemical study of the case, as well as of bone marrow biopsy and CT-scan, renders diagnosis not confirmed. As far as primary testicular T-LBL is concerned, no previous case has been reported.

Due to the morphology of neoplastic cells (small to medium-sized blast cells with scant cytoplasm, moderately condensed to dispersed nuclear chromatin and indistinct nucleoli to larger blasts with finely dispersed nuclear chromatin and relatively prominent nucleoli), differential diagnosis include both lymphoid and non lymphoid neoplasm, as BL, DLBCL, blastoid variant of MCL, small lymphocytic lymphoma, B1 thymoma, myeloid sarcoma, small cell carcinoma, ES/PNET, rhabdomyosarcoma, seminoma, Merkel cell carcinoma [1,6,7]. A challenging differential diagnosis, especially in younger patients, is with all the tumors belonging to the so-called small round blue cell tumor category [1,2,6,7]. The age of the patient, the clinical presentation, a careful morphologic examination and, finally, immunophenotyping generally permit distinction among these diagnostic entities. In the uterine case, the differential diagnosis among DLBCL, BL, myeloid sarcoma, blastoid variant of MCL, small lymphocytic lymphoma, small (oat) cell carcinoma, rhabdomyosarcoma and PNET was proposed (Table 1). However, an immunohistochemical panel including lymphoid, myeloid, epithelial and mesenchimal markers, ruled out any doubts. In the testicular case, the differential diagnosis among seminoma, BL, DLBCL, myeloid sarcoma, PNET, neuroblastoma, and embryonal rhabdomyosarcoma was made (Table 1). Also in this case, immunohistochemistry was helpful in achieving diagnosis. However, the ambiguous lineage of neoplastic cells induced us to perform TCR and immunoglobulin heavy chain gene (IgH) rearrangement to confirm the final diagnosis. It is noteworthy that patients with T-cell LBL often do express antigens more commonly associated with other lineages, including B-cell associated antigens CD10, CD20 and CD79a [6] as a consequence of an initial oncogenic hit affecting the hematopoietic stem cell or a common lymphoid precursor element. Therefore, in a minority of cases, lineage assignment on the basis of immunophenotype still remains unclear despite the application of a suitable panel of markers, thus molecular biology analysis is necessary. Although, Pax-5 completely arrests T-cell development, its expression has been reported in some mature T-cell lymphomas but not in T-cell LBL [17]. Nonetheless, experimental evidence have shown that aberrant Pax-5 expression in thymocytes drives malignant transformation [18]. These findings suggest that Pax-5 may play a role in T-cell lymphomagenesis, including T-LBL.

**Table 1:** Differential diagnosis of uterine and testicular T-cell lymphoblastic lymphoma

**Neoplasm**	**Clinical features**	**Morphology**	**Immunophenotype**	**Molecular features**
**B-LBL**	median age 20 years, M > F, 90% of cases present as B-ALL	convoluted or round nuclear contours; immature blastic chromatin; numerous mitotic figures	CD19 +, PAX-5 +, CD20 +/−, TdT +, CD10 +/−, CD79 + (in 10% of cases), T-cell antigens -	monoclonal IgH gene rearrangement; t (9; 22) (q34; q11.2); t (12;21) (p13; q22); t (v; 11q23); t (1; 19) (q23; p13.3); t (5; 14) (q31; 32)
**BL**	childhood; head and neck and ileocecal region frequently involved	prominent “starry sky” pattern; monotonous, medium-sized cells with 2–5 prominent nucleoli and distinct cytoplasmic rim; very high mitotic and apoptotic rates	CD10 +, CD19 +, CD20 +, CD79a +, Bcl2 -, Bcl6 +, TdT -, Ki-67 > 99%, T-cell antigens -	monoclonal IgH gene rearrangement; t (8; 14) (q24; q32) or t (2; 8) (p11; q24) or t (8; 22) (q24; q11) involving *MYC*
**DLBCL**	adult; frequent nodal involvement	large or medium-sized cells with prominent nucleoli; centroblastic or immunoblastic appearance	CD19+, CD20+, CD79a+, PAX-5+, CD10 +/−, Bcl6 +/−, IRF4/MUM1 +/−, Bcl2 +/−, TdT -, T-cell antigens -	t (14; 18) (q32; q21); Bcl6 (3q27) rearrangement; MYC (8q24) rearrangement
**Myeloid sarcoma**	median age; frequent history of previous or concomitant AML, MDS, MPN, MDS/MPN	myelocytes with more distinct and eosinophilic cytoplasm	CD43 +, CD68PGM1 +, CD68 +, CD117 +, MPO +, CD10 -, T-cell antigens -	no evidence of monoclonal TCR gene rearrangements, monosomy 7, trisomy 8
**Blastoid variant of MCL**	median age 68 years, male predominance; frequent extranodal involvement	lymphoblastoid cells with immature chromatin and high mitotic rate	CD19 +, CD20 +, Cyclin-D1 +, CD5 +, CD10 -, TdT -, CD2 -, CD3 -, CD7 -	monoclonal IgH gene rearrangement; no evidence of monoclonal TCR gene rearrangements; t (11; 14) (q13; q32)
**ES/PNET**	median age <20 years, M > F; frequent bone involvement	cohesive growth pattern, frequently pseudorosettes formation; small blue monomorphic round cells with fine nuclear chromatin, round nuclei and scanty clear cytoplasm	CD99 +, vimentin +, WT-1 +, lymphoid markers -	no evidence of monoclonal TCR gene rearrangement; t (11; 22) (q24; q12) or t (21; 22) (q22; q12) or t (1; 16) (q11; q11)
**Alveolar rhabdomyosarcoma**	adolescents and young adults; extremities and paraspinal region frequently involved	nests of round cells separated by fibrous septa, with some giant cells and occasional clear cells	vimentin, desmin, smooth muscle actin, HHF-35, MyoD1, myogenin +, cytokeratins +/−, S100 +/−, CD20 +/−, T-cell antigens -, TdT-	t (2; 13) (q35; q14) or t (1;13) (p36; q14)
**Small (oat) cell carcinoma**	middle age; frequent pulmonary invlvement	small ovoid, round to spindled cells with markedly increased nuclear-to-cytoplasmic ratio, hypercromatic nuclei and inconspicuous nucleoli	low molecular weight keratins +, chromogranin +/−, synaptophysin +/−, CD3 -, CD5 -, TdT -	
**Seminoma**	young adults; testicular enlargement and hydrocele	round, polygonal cells with large and vesicular nuclei, prominent nucleoli, clear and eosinophilic cytoplasm and frequent mitoses, lymphoid infiltrate in the backgournd	CD117 +, D2-40 +, OCT4 +, SALL4 +, cytokeratins -, CD30 -, EMA -, T-cell antigens -, TdT -	

B-LBL: B lymphoblastic lymphoma; BL: Burkitt lymphoma; DLBCL: diffuse large B-cell lymphoma; AML: acute myeloid leukemia, MDS: myelodysplastic syndrome; MPN: myeloproliferative neoplasm; MDS/MPN: myelodysplastic/myeloproliferative syndrome; MCL: mantle cell lymphoma; ES: Ewing sarcoma; PNET: peripheral neuroectodermal tumour; +: positive; −: negative; IgH: immunoglobulin heavy chain gene; TCR: T-cell receptor gene.

Once diagnosis of T-LBL has been confirmed, a complete staging is required to exclude bone marrow or other organ involvement, as in our cases. T-LBL is a clinically aggressive disease with a high risk of induction failure, frequent relapse and poor survival [1]. Accordingly, high dose combined systemic and intrathechal chemotherapy, followed by intensive consolidation treatment, improves prognosis, especially in young adults [5]. Hematopoietic SCT produces favorable long-term outcome in selected adult patients [4]. Our patients were both treated by the hyper-CVAD protocol associated to intrathecal prophylaxys. The female patients did not underwent treatment with high-dose cytarabine and methotrexate as it has been yielded no clear benefits in older patients [3]. Only the patient with testicular lymphoma is alive at the last follow-up, perhaps for his younger age.

## Conclusions

We reported two patients (ageing respectively 64 and 38 years) with T-LBL presenting as an uterine and testicular mass. Only one doubtful previous case of primary uterine T-LBL and no previous cases of primary testicular T-LBL have been described so far. Although the frequency of this type of lymphoma at these sites is very low, primary T-LBL lymphoma still needs to be considered in the differential diagnosis of diffuse small blue cells proliferation. Molecular biology may represent an adequate tool to confirm diagnosis in ambiguous lineage cases. Unfortunately, no clear prognostic factors that may predict remission or survival are well established for T-LBL [1]. Minimal residual disease detection is one of the strongest predictors of relapse risk [6]; however, identification of other clinical, biological and radiological parameters is critical for risk stratification, especially in adult patients, to select those who may benefit of SCT [1].

## Consent

Written informed consent was obtained from the patients for publication of this Case Report and any accompanying images. A copy of the written consent is available for review by the Editor-in-Chief of this journal.

## Abbreviations

TdT: Terminal deoxynucleotidyl transferase; ALLs: Acute lymphoblastic leukemias; LBL: Lymphoblastic lymphomas; CNS: Central nervous system; SCT: Stem cell transplantation; CVAD: Cyclophosphamide, vincristine sulfate, adryamicin, dexamethasone; BL: Burkitt lymphoma; DLBCL: Diffuse large-B cells lymphoma; ES: Ewing sarcoma; TCR: T-cell receptor; PNET: Peripheral neuroectodermal tumor; IgH: Immunoglobulin heavy chain.; TCR: T-cell receptor; MCL: Mantle cell lymphoma; MDS: Myelodysplastic syndromes; MPN: Myeloproliferative neoplasm.

## Competing interests

The authors declare that they have no competing interests.

## Authors’ contributions

MRA wrote the paper; MO, BJR and GP evaluated the immunoassays; AG and GL made contributions to acquisition of clinical data; ADV supplied information on the therapeutic approach; FDN and RS contributed their expertise in the field and fruitful discussion; SL gave final approval of the version to be published. MRA and SL coordinated the work. All authors read and approved the final manuscript.
